# Phyto-polyphenols as potential inhibitors of breast cancer metastasis

**DOI:** 10.1186/s10020-018-0032-7

**Published:** 2018-06-05

**Authors:** Dimiter Avtanski, Leonid Poretsky

**Affiliations:** 1 Gerald J. Friedman Diabetes Institute at Lenox Hill Hospital, Northwell Health, New York, NY 10022 USA; 2 Division of Endocrinology and Metabolism, Department of Medicine, Friedman Diabetes Institute at Lenox Hill Hospital, Northwell Health, 110 E 59th Street, Suite 8B, Room 837, New York, NY 10022 USA

**Keywords:** Polyphenols, Breast cancer, Metastasis, Plant products, Resveratrol, EGCG, Kaempferol

## Abstract

Breast cancer is the most common cancer among women as metastasis is currently the main cause of mortality. Breast cancer cells undergoing metastasis acquire resistance to death signals and increase of cellular motility and invasiveness.

Plants are rich in polyphenolic compounds, many of them with known medicinal effects. Various phyto-polyphenols have also been demonstrated to suppress cancer growth. Their mechanism of action is usually pleiotropic as they target multiple signaling pathways regulating key cellular processes such as proliferation, apoptosis and differentiation. Importantly, some phyto- polyphenols show low level of toxicity to untransformed cells, but selective suppressing effects on cancer cells proliferation and differentiation.

In this review, we summarize the current information about the mechanism of action of some phyto-polyphenols that have demonstrated anti-carcinogenic activities in vitro and in vivo. Gained knowledge of how these natural polyphenolic compounds work can give us a clue for the development of novel anti-metastatic agents.

## Background

Breast cancer is the most common cancer in women, accounting for nearly 1 in 3 diagnosed cancers or 16% of all female cancers. The incidence of breast cancer increases with age and is expected to escalate due to the increase in life expectancy and the adoption of the Western lifestyle and rising rates of obesity. In spite of the advances in treatment, metastasis remain the main cause of mortality in cancer patients contributing to 90% of deaths from solid tumors (Gupta & Massagué 2006).

Natural products are used in traditional medicine over the millennia for prevention and treatment of variety of maladies, including cancer. Plants are rich in polyphenolic compounds and many of these compounds have proven beneficial effects in preventing the initiation and development of metastasis. Natural polyphenols have generally pleiotropic effects in the cell, activating multiple signaling pathways thus affecting many aspects of cellular fate, including cell apoptosis, proliferation, and differentiation. In this regard, it is worth studying the mechanism of action of the natural polyphenols which can give us clues for the development of new synthetic therapeutic molecules.

In this review, we summarize the main in vitro and in vivo effects of some promising phyto-polyphenols that have shown suppressing actions in the initiation and progression of metastasis in breast cancer. Some of these polyphenolic compounds are already in phase I, II, or III clinical trials.

## Breast cancer and metastasis

### Epidemiology of breast cancer and metastasis

According to the American Cancer Society, the average age at the time of breast cancer diagnosis is 61 years. Although, breast cancer predominates in women, about of 1% of all cases occur in men. Among the different ethnicities, breast cancer incidence rates are higher in non-Hispanic Caucasian women compared to African American women, but mortality rates are higher among African Americans (32%) compared to non-Hispanic Caucasians (24%). The most recent data for American women diagnosed with breast cancer demonstrate survival rates of 89, 82, and 77% at 5, 10, and 15 years after diagnosis (American Cancer Society, 2011).

Human breast cancer is a heterogenous disease, which can be classified into different groups depending on the presence or absence of estrogen receptor (ER), progesterone receptor (PR), and human epidermal growth factor receptor 2 (HER2) expression. The expression of these three receptors strongly defines breast cancer behavior and treatment options. For example, HER2-positive breast cancers are more aggressive in nature, but respond better to the current therapy resulting in more favorable prognosis. Much more challenging are the triple-negative breast cancers (TNBC) (ER/PR/HER2-negative), constituting between 10 and 20% of all breast cancers, which are characterized by most aggressive behavior and lack effective therapies.

The prognosis in breast cancer strongly depends on the presence or absence of metastasis in other organs. Today, around 155,000 people in the United States live with metastatic breast cancer and approximately 6–10% of all newly diagnosed breast cancer patients are present with metastatic disease at the time of diagnosis (American Cancer Society, 2011) (NCI SEER 2018). Cancer metastases occur in 20–30% of all breast cancer cases and the median survival of metastatic breast cancer patients is on average 3 years (O’Shaughnessy 2005).

### Mechanism of breast cancer metastasis

Metastasis is a process involving interplay between the cancer cells with their biological properties and the host distant site providing specific microenvironment. Particular tumors have the affinity to spread in particular organs. In 1889 Stephen Paget formulated the so called *“seed and soil”* theory, which is based on autopsy records of 735 women with breast cancer (Paget 1989). According to this theory, the *‘seed’* is the metastatic cell, and the *‘soil’* the metastatic site. The basic idea behind the Paget’s theory is that in order to metastasize, cancer cell must find a suitable location bearing certain characteristics. Later, in 1928, the American pathologist James Ewing challenged the *“seed and soil”* theory, suggesting that the organ specific metastases could be explained by pure anatomical and mechanical circulatory patterns between the primary tumor and the distant organs (Ewing 1928). In fact, the compatibility between the cancer cells and the host environment as well as the circulatory patterns play roles in the metastatic process. The determination which organ would be a target for cancer invasion depends on the proximity of the tumor side to the host organ and the connection between the primary tumor and the metastatic site through the vascular circulatory system. For example, breast cancer commonly metastasizes to bones or the ovaries. In addition to using blood vessels, cancer cells (e.g. breast carcinoma cells) can migrate by invading the lymph nodes and using the lymphatic system, but they ultimately rely on the blood vessels to find their way to the distant site.

To be able to metastasize the cancer cell must undergo physiological changes and overcome numerous obstacles. Generally, the metastatic process could be divided into several defined stages: (1) loss of cellular adhesion, (2) increase of cellular motility and invasiveness, (3) entry and survival in the circulation, (4) spread into distant tissue, and (5) colonization of the distant site (Chambers et al. 2002). At the beginning of the metastatic process, the primary tumor needs to develop its own blood circulatory system which also provides a route for tumor migration. Progression toward metastasis requires acquiring a resistance to cell death signals accomplished by overexpression of anti-apoptotic effector genes such as B-cell lymphoma 2 (BCL2), BCL-XL, and X-linked inhibitor of apoptosis protein (XIAP) (Mehlen & Puisieux 2006). Cancer cells undergoing metastasis are characterized by increased expression of matrix metalloproteinases (MMPs), which proteolytically disrupt the protective basal membrane (MacDougall & Matrisian 1995). Secreted proteases generate a variety of bioactive cleavage peptides which further modulate cancer cell migration, proliferation, survival, and tumor angiogenesis (Gupta & Massagué 2006). Once the cancer cells enter the bloodstream, they increase the secretion of proteins such as autocrine motility factor (AMF) and motility-stimulating protein (MSP) which enable them to survive the harsh conditions in the bloodstream (Watanabe et al. 1991). Finally, the cancer cells extravasate from the circulation and enter the new site where they form pre-angiogenic micrometastases (Chambers et al. 2002).

Underlying event in metastasis is the epithelial-to-mesenchymal transition (EMT), a process in which particular cells lose their epithelial characteristics and gain more mesenchymal-like features. During EMT the cellular expression of cell adhesion molecules (CAMs) decrease resulting in the formation of spindle-shape morphology. EMT is a fundamental process occurring during the embryonal development (designated as Type I EMT), fibrosis or wound healing (or Type II EMT), but EMT also plays a key role in cancer metastasis (also known as Type III EMT) (Kalluri & Weinberg 2009). Main event during EMT is the cleavage of the tight junction cell surface protein E-cadherin and inhibition of its expression by SNAIL, SLUG, ZEB and TWIST transcription factors accompanied by overexpression of N-cadherin, fibronectin, vimentin and other proteins (Peinado et al. 2007; Yang & Weinberg 2008). Cancer cells involved in EMT undergo dynamic cytoskeletal rearrangements interacting intensively with the cell-matrix. This process is governed by growth factors, which directly or indirectly modulate plasma membrane proteases and focal adhesion disassembly (Gupta & Massagué 2006).

Migratory cancer cells show elevated expression MMPs, which are Calcium-dependent Zinc-containing endopeptidases capable of degrading extracellular matrix (ECM) proteins (Verma & Hansch 2007). There is a strong correlation between the MMP expression and cancer invasion and metastasis (Kanadaswami et al. 2005). MMPs participate in all stages of carcinogenesis and are particularly important for tumor invasion (McCawley & Matrisian 2000). Generally, overexpression of MMPs is linked to higher metastasis capacity in many tumors (Kanayama 2001; Saito et al. 2007; Castellano et al. 2008; Lee et al. 2008). Expression of MMPs is induced by growth factors (like epithelial growth factors [EGFs]) and receptor tyrosine kinase (RTKs) (such as EGF receptor [EGFR]) involving PI3K (phosphatidylinositol-3-kinase) and NF-κB (nuclear factor kappa-light-chain-enhancer of activated B cells) signaling cascades (Sen & Chatterjee 2011). Experimental results have shown that inhibition of MMPs results in abolishment of tumor cell invasiveness (Matrisian 1990; Rhee & Coussens 2002; Van den Steen et al. 2002; Kanadaswami et al. 2005). For this reason, MMPs are considered as important molecular targets for the anticancer therapy. Among the 23 currently known human MMPs, the gelatinases (also known as type IV collagenases) MMP-2 (*s.* gelatinase A) and MMP-9 (*s.* gelatinase B) play key roles in the metastatic process. MMP-2 and -9 are suppressed by tissue inhibitors of metalloproteinases (TIMPs) (Visse & Nagase 2003). There are four different TIMPs, TIMP1, 2, 3, and 4, which bind non-covalently to MMP thus inhibiting their expression (Brew & Nagase 2010).

NF-κB is a main player in the metastatic process because it is crucial regulator of cell proliferation and survival. NF-κB levels may predict the potential of the tumor cells to metastasize (Jin et al. 2014). In resting cells NF-κB exists in inactive form, located in the cytoplasm, bound to a family of inhibitory proteins referred as IκB (inhibitors of κB). Members of IκB family include IκB-α, IκB-β, IκB-γ, IκB-ε, IκB-ζ, p105, p100, and bcl3, as IκB-α (also known as nuclear factor of kappa light polypeptide gene enhancer in B-cells inhibitor-alpha [NFKBIA]) is the most abundant among them. The control of NF-κB activity is carried out by IκB kinase (IKK) kinases which include mitogen-activated protein kinase kinase (MAPKK) family comprising of NF-κB-inducing kinase (NIK) and MAPK/ERK kinase kinase (MEKK) 1, 2, and 3. When activated, NF-κB translocates to the nucleus where it serves as transcription factor regulating genes controlling cell cycle, apoptosis, transformation, and other processes. Constitutively active NF-κB is characteristic for many cancers. It protects the activation of apoptotic signal by inhibiting p53 activity thus promoting the survival and neoplastic transformation of the cancer cells. The NF-κB signaling induces the expression of a number of target genes involved in angio- and lymphangiogenesis among them the vascular endothelial growth factor (VEGF). NF-κB directly induces the expression of urokinase-type plasminogen activator (uPA) (Sliva et al. 2002), MMP-9, and chemokine receptor CXCR4 (Helbig et al. 2003), which in turn results in promotion of ECM degradation and metastasis. The regulation of tumor metastasis by NF-κB is exerted by reciprocal regulation of prometastatic (heparanase, etc.) and antimetastatic (MMP-1, MMP-2, plasminogen activator inhibitor [PAI]-2, etc.) factors. Thus NF-κB is considered as an attractive candidate for metastasis treatment. Number of developed therapeutic agents aim to target NF-κB activity and function by different approaches such as induction of IκBα expression or prevention of its degradation, inhibition of NF-κB nuclear translocation, suppression of NF-κB binding to DNA, inhibition of IKK functions, etc. (Wu & Kral 2005)

It is widely accepted that tumors are initiated by small proportion of cancer stem cells (CSCs) that possess capacity for indefinite self-renewal. CSCs bear CD44^+^/CD24^−/low^ lineage characteristics and differentiate into all other cellular phenotypes in the solid tumor as well as they can initiate the formation of secondary tumors. Recent experimental results suggest that microRNAs (miRs) play a critical role in the formation of CSCs and the acquisition of EMT (Li et al. 2010).

### Role of tumor microenvironment in breast cancer metastasis

Tumor is a complex structure comprised not only by the neoplastic cells, but also by other cellular types of a different origin, all of them residing in a specific ECM microenvironment and communicating via soluble substances (Yu & Di 2017). Tumor-infiltrating lymphocytes (TILs) are component of the tumor microenvironment that play a major role in cancer development. Most of the TILs are CD8^+^ T cells, CD4^+^ helper T cells (Th), and CD4^+^ regulatory T cells (Tregs), as evidence suggest that TILs are predictor of tumor outcome (Haanen et al. 2006). Huang et al. (2015) demonstrated that although both, CD8^+^ and CD4^+^ cells have a role in cancer, during breast cancer development the number of Th cells increase concomitantly with a change of their dominant subsets from Th1 to Treg. On the other hand, CD8^+^ cells are inverse indicator of ER and PR status in the breast tumor and may predict the clinical outcome (Mahmoud et al. 2011). Another component of the tumor microenvironment are the tumor-associated macrophages (TAMs) which are monocytes recruited by cytokines (such as the chemokine (C-C motif) ligand 2 [CCL2]) from the peritumoral tissues or bone marrow. TAMs can be divided into M1 and M2 machrophages, but studies also suggest that they may actually possess characteristics of both (Yu & Di 2017). Driven by interleukin (IL)-4 and IL-10, tumor necrosis factor-alpha (TNFα), macrophage colony-stimulating factor (M-CSF), or hypoxia, breast tumor microenvironment facilitate M1 differentiation into M2 (Laoui et al. 2011). Hypoxia of the white adipose tissue may be induced by obesity and can further lead to endocrine alterations promoting the secretion of proinflammatory and angiogenic cytokines, and downregulating CCAAT-enhancer binding protein-alpha (C/EBPα) thus inhibiting apoptosis and stimulating cell proliferation (Ye et al. 2007; Khan et al. 2013). Since the cytokines released by the M1 macrophages in the early stages of cancer development have anti-proliferative effects on tumor cells, the increased proportion of M2 macrophages in the later stage of tumor development facilitate cancer growth (Quail & Joyce 2013). Cancer-associated fibroblasts (CAFs) are other component of the tumor microenvironment. It is suggested that these cells have heterogeneous origin and derive from neighboring tissue fibroblasts, bone marrow mesenchymal cells, epithelial cells undergoing EMT or other cellular types (Shiga et al. 2015). CAFs directly modulate tumor progression and metastasis by secreting growth factors and cytokines that promote ECM remodeling, cellular proliferation, EMT, and angiogenesis (Cirri & Chiarugi 2011). Adipocytes are main component of the mammary gland. In human, fat volume comprises an average of 25% (7–56%) (Vandeweyer & Hertens 2002) of the non-lactating and an average of 35% (9–54%) (Ramsay et al. 2005) of the lactating breast tissue. Mammary adipose cells share characteristics with the subcutaneous WAT adipocytes, but are distinctive from these cells by their response to menstrual cycle and permanent interactions with the surrounding epithelial cells (Choi et al. 2017). Adipose cells are also a major component of the tumor microenvitonment and are especially prominent in the breast tumors. Cancer-associated adipocytes (CAAs) are smaller than the non-tumor-associated adipocytes and are highly secretory cells reprogrammed by the tumor cells into dedifferentiated preadipocyte stage. The role that CAAs play in tumor development is supported by epidemiological observations of higher breast cancer incidence in obese postmenopausal women (Calle & Kaaks 2004) and associations of obesity with poorer clinical outcome (Reeves et al. 2007; Chan et al. 2014). CAAs affect cancer cells proliferation, survival and invasion potential by secreting various adipokines, lipids and reactive oxygen species (ROS) thus provoking ECM remodeling and metabolic transformations (Choi et al. 2017; Nieman et al. 2013; Berstein et al. 2007). Another component of the tumor microenvironment are the endothelial cells (tumor endothelial cells [TEC]). These cells differ from the normal epithelial cells in their responsiveness to EGF, VEGF and other growth factors, and are associated with tumor cells adhesion, invasion, and metastasis (Hida et al. 2013). Besides of the cellular components, ECM by itself plays a multifaceted role in tumor development through biochemical and biomechanical mechanisms (Yu & Di 2017).

## Phyto-polyphenols with promising inhibitory effects on breast cancer metastasis

Polyphenols (*s.* polyhydroxyphenols) are class of chemical compounds, broadly distributed in nature and characterized by the presence of phenol structures in their molecules. A vast group of polyphenols universally present in the plant kingdom is the bioflavonoids. Comprising more than 4000 distinct members, bioflavonoids are 15-Carbon skeleton derivatives of beno-γ-pyrone (*s.* phenylchromone). Flavonoids are divided into different classes that include flavonols, glavans and proanthocyanidins, anthocyanidins, flavanones, flavones, isoflavones, and noeflavonoids.

Phyto-polyphenols are integral part of the human diet. They have been also used worldwide in traditional medicine for thousands of years for their anti-bacterial, anti-viral, anti-inflammatory, anti-allergic, and anti-thrombotic properties. The effects of phyto-polyphenols are usually pleiotropic, and many of these compounds have proven anti-carcinogenic actions manifested by suppression of cancer cell transformation, differentiation, proliferation and invasiveness, angiogenesis and induction of apoptosis. The anti-carcinogenic properties of the phyto-polyphenols can be attributed to their direct effects on the activities of key protein kinases controlling tumor cell proliferation and apoptosis or to the suppression of MMP function. For example quercetin, fisetin or luteolin and other phyto-polyphenols inhibit the activity of protein kinase C (PKC). PKC plays an important role in a variety of processes in cancer, from tumor initiation and progression to inflammation and T lymphocyte function. Genistein (Akiyama et al. 1987; Peterson & Barnes 1991; Pagliacci et al. 1994), luteolin (Huang et al. 1999; Lee et al. 2002), quercetin (Agullo et al. 1997), and butein (Yang et al. 1998) affect tumor development by suppressing the activity of epidermal growth factor receptor (EGFR) tyrosine kinase resulting in downstream effects on number of substrates such as serine/threonine kinases, mitogen-activated protein kinases (MAPKs), and rapidly accelerated fibrosarcoma kinases (RAFs) (Carpenter & Cohen 1990). Another protein tyrosine kinase that is targeted by phyto-polyphenols (luteolin, quercetin, etc.) is the focal adhesion kinase (FAK) (Kanadaswami et al. 2005). FAK is a key molecule in signaling pathways essential for the cell cycle, survival, and motility.

Whole extracts or specific polyphenols derived from green tea or grape vines have been shown to possess anti-carcinogenic and anti-metastatic properties in multiple in vitro and in vivo studies. Extracts from peach (*Prunus persica*) (Noratto et al. 2014), olive (*Olea europaea*) (Hassan et al. 2012), promegranate (*Punica granatum*) (Kim et al. 2002), evening primrose (*Oenothera paradoxa*) (Lewandowska et al. 2013a; Lewandowska et al. 2013b), the spotted (*s.* prostrate) spurge (*Euphorbia suprina, (s. E. maculata)*) (Ko et al. 2015), Japanese quince (*Chaenomeles japonica*) (Lewandowska et al. 2013c), Himalayan rhubarb (*Rheum emodi*) (Kumar et al. 2015; Naveen Kumar et al. 2013) or *Phyllanthus sp.* (*P. niruri, P. urinaria, P. watsonii, P. amarus*) (Lee et al. 2011), and others inhibit tumor growth and suppress breast cancer metastasis.

### Grape polyphenols

Grape vine plant consists of three main species: the European grapes (*Vitis vinifera*), the North American grapes (*V. lanrusca* and *V. rotundifolia*), and French hybrids. Grape vines belong to the *Vitaceae* family and were domesticated as early as in the Neolithic period. Grapes contain variety of polyphenolic compounds largely anthocyanins, flavonols (catechin, epicatechin, quercetin, procyanidin polymers), stilbenes (resveratrol), and phenolic acids. Grape polyphenols are distributed mostly in the seed, skin, leaf and the stem of the plant, and in considerably less amount in its juicy middle section. Resveratrol, quercetin and catechin polyphenols represent about 70% of the polyphenols present in the grape plant and have the most potent anti-carcinogenic activities (Damianaki et al. 2000). Importantly, grape polyphenols are easily absorbed and metabolized in the body in their intact form (Soleas et al. 2002). Experimental data demonstrate that grape polyphenols have cardio- and neuro-protective, anti-microbial (Lagneau et al. 1998; Xia et al. 2010; Castillo-Pichardo et al. 2013), anti-oxidant (Torres et al. 2002; Negro et al. 2003; Makris et al. 2007) and variety of anti-carcinogenic (anti-proliferative, pro-apoptotic, anti-invasive, anti-angiogenic, antioxidant, and cancer-preventive) properties (Soleas et al. 2002; Asensi et al. 2002; Nifli et al. 2005; Morré & Morré 2006; Hakimuddin et al. 2008; Gulati et al. 2006; Kim et al. 2004; Dechsupa et al. 2007; Aggarwal & Shishodia 2006; Kaur et al. 2009).

The suppressing effects of the grape polyphenols on breast cancer initiation and cell growth are demonstrated in multiple in vitro and in vivo systems (Singletary et al. 2003; Hakimuddin et al. 2004) (Singh et al. 2004) (Schlachterman et al. 2008). Using nude mice xenografted with GFP-tagged highly metastatic ER-negative MDA-MB-468 breast cancer cells, Castillo-Pichardo et al. (2009) found that low concentrations of grape polyphenols can inhibit breast cancer metastasis initiation, specifically to liver and bone. Experiments using BALB/c 4 T1 mammary xenograft mouse model showed that treatment with dietary grape skin extracts in drinking water resulted in decrease of lung metastasis incidence and stimulate cell survival (Sun et al. 2012). Resveratrol, quercetin and catechin are particularly important in estrogen receptor (ER)-positive breast tumors since they also act as selective estrogen receptor modulators (SERMs) (Harris et al. 2005). Grape polyphenols exert their effects by modulating the activities of Akt, extracellular-signal-regulated kinases (ERKs), and MAPKs (Lu et al. 2009; Kaur et al. 2011; Sun et al. 2012). These polyphenols inhibit the expression and activity of EGFR1 and EGFR2 (*s.* HER2) (Azios & Dharmawardhane 2005; Fridrich et al. 2008), and elevated EGFR tumor expression is generally associated with higher cancer progression and metastasis (Buret et al. 1999). HER2 plays a major role in the metastatic process and its overexpression is often observed in metastatic cancers. Inhibition of HER2 by grape polyphenols leads to inhibition of phosphatidylinositol-3-kinase (PI3K)/Akt and mammalian target of rapamycin (mTOR) as well as activation of 5’ AMP-activated protein kinase (AMPK) – all of these enzymes are involved in the process of metastasis. Additionally, grape polyphenols upregulate forkhead box O1 (FOXO1) and IκBα thus inhibiting NF-κB activity (Castillo-Pichardo et al. 2009).

#### Resveratrol

**Fig. 1:**
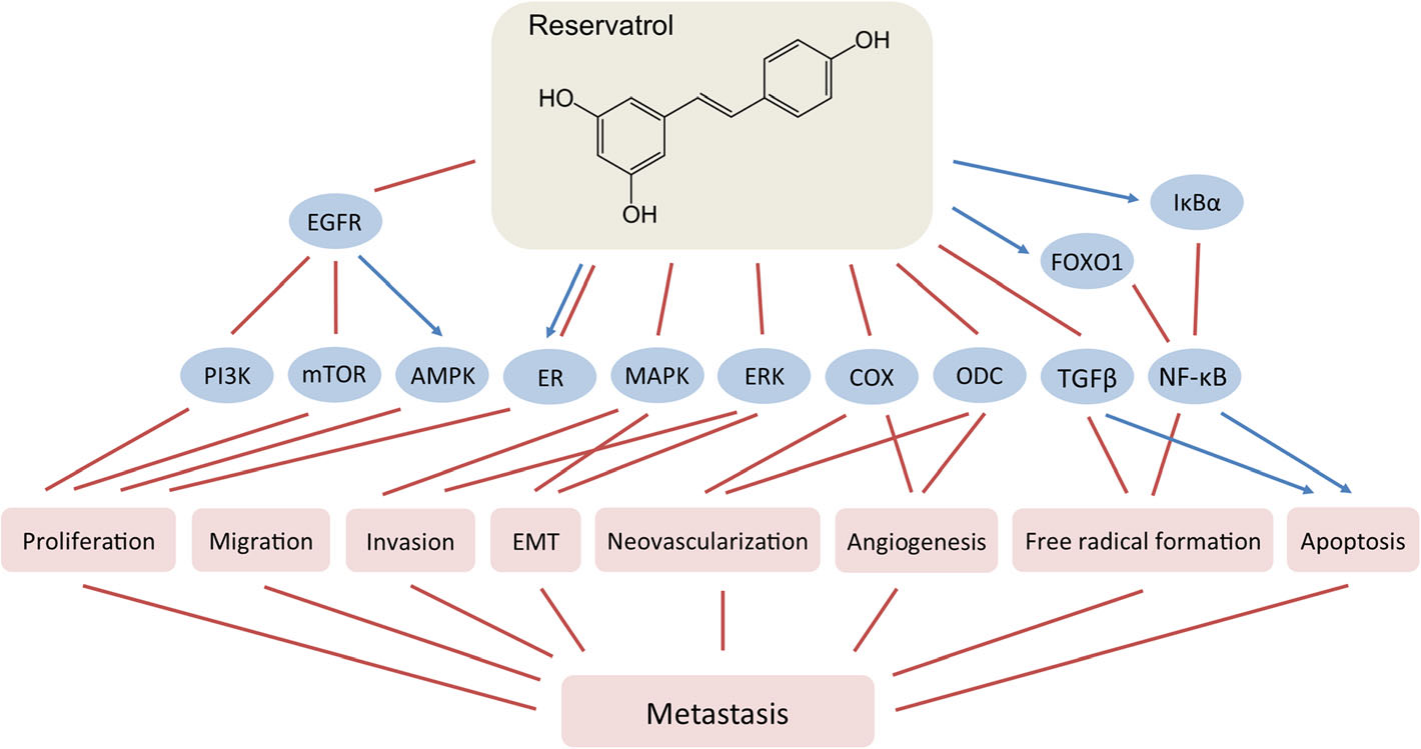
Effects of reservatrol on breast cancer metastasis

Resveratrol (3,5,4′-trihydroxy-*trans*-stilbene) is a stilbenoid and phytoalexin produced by grapes, peanuts, berries, and the Japanese “Kojokon” (*Polygonum cuspidatum*) in response to injury or pathogen invasion (Burns et al. 2002). Chemically, resveratrol is a precursor of a family of polymers named viniferins. It quickly enters the bloodstream from the gastro-intestinal tract, reaching significant plasma concentrations (Bhat et al. 2001). Resveratrol has been used for centuries in the traditional Asian medicine since it has broad range of effects, including anti-oxidant properties, modulation of lipid and lipoprotein metabolism, anti-platelet aggregation, vaso-relaxation, wound-healing, estrogenic activities and multiple anti-carcinogenic effects. The anti-carcinogenic properties of resveratrol have been demonstrated in many types of cancer including those of the breast (Fig. 1) (Delmas et al. 2006; Busquets et al. 2007; Castillo-Pichardo et al. 2009). They include tumor cell proliferation arrest, induction of apoptosis, suppression of tumor cell mobility and migration, prevention of tumor-derived nitric oxide synthase expression, inhibition of tumor progression, etc. (Jang et al. 1997; Nakagawa et al. 2001; Garvin et al. 2006) Resveratrol is a SERM that acts in different tissues as a pro- or anti-estrogen (Bowers et al. 2000).

Current literature exploring the in vivo doses of resveratrol needed to achieve beneficial anti-carcinogenic effect is still not consolidated. In fact, low doses of reservatrol achievable from dietary sources (such as red wine) seem to be sufficient in suppressing tumor growth (Tessitore et al. 2000). Resveratrol might be an effective chemopreventive agent and the mechanism behind this effect includes direct inhibition of cyclooxygenase (COX) activity and indirect suppression of ornithine dacarboxylase (ODC) (Jang et al. 1997; Subbaramaiah et al. 1999; Baur & Sinclair 2006). The effect of resveratrol on COX and ODC activities could also explain its anti-neovascularization and anti-angiogenic properties.

In vitro and in vivo studies have showed that resveratrol inhibits NF-κB and decreases its DNA binding resulting in modulation of transcription of genes involved in tumor growth and metastasis (Tsai et al. 1999; Banerjee et al. 2002; Benitez et al. 2009). Results from a study using Sprague-Dawley rats where resveratrol was given in the diet two weeks before vein injection with the tumor-initiating agent 7,12-dimethylbenz(a)anthracene (DMBA) demonstrated that resveratrol acts as a strong antioxidant and significantly induces apoptosis with concomitant upregulation of TGFβ1 expression and inhibition of NF-κB in these carcinogen-challenged animals (Chatterjee et al. 2011). Experiments using female FVB/N HER2/*neu* transgenic mice spontaneously developing mammary tumors revealed significant reduction of lung metastases incidence after oral resveratrol supplementation (Provinciali et al. 2005). Contrary to the previous results, resveratrol was found to promote tumor growth and metastases incidence in immunocompromised mice grafted with low-metastatic ERα-negative/ERβ-positive MDA-MB-231 or highly-metastatic ERα/ERβ-negative MDA-MB-468 breast cancer cells (Castillo-Pichardo et al. 2013). The reason for the discrepancy between the experimental in vivo data may be explained with the different protocols followed for drug administration, the variable concentration of reservatrol used or combination of multiple other factors.

Besides acting on tumor cells, reservatrol have modulating effects on tumor microenvironment. It induces CD8^+^ T cells antitumor immunity, decreases the percentage of Tregs in the tumor, increases the levels of interferon-gamma (IFNγ) and reduces those of IL-6, IL-10, and VEGF, as shown in renal tumor model (Chen et al. 2015). Reservatrol also reduces oxidative stress by acting as a direct scavenger of ROX, by inhibiting NADPH oxidase expression or xanthine oxidase activity (Pelicano et al. 2004; Lin et al. 2000), or by increasing sirtuin 1 (SIRT1) activity (Xu et al. 2012).

In summary, although the anti-carcinogenic and cancer-preventive properties of resveratrol are proven in multiple studies, the real efficacy of this compound in vivo is still unclear. The clinical evidence for resveratrol as an effective supplement for cancer prevention and treatment is scarce as at this time there is very little clinical data for the efficacy of resveratrol in cancer treatment.

### Green tea polyphenols

Green tea is a product of leafs and the leaf buds of *Camellia sinensis* plant that belongs to *Theacea* family. Green tea contains more than 200 bioactive compounds, among them polyphenols (catechins and flavonols), alkaloids (caffeine), amino acid analogs (theanine), vitamins, minerals, etc. Polyphenols are the largest and most active group of chemical compounds in the green tea comprising about 40% of the leave dry weight. Polyphenols found in green tea include: epigallocatechin-3-gallate (EGCG) (48.6%), epicathechin gallate (ECG) (12.3%), epigallocatechin (EGC) (4.1%), epicatechin (EC) (4.1%), gallocatechin gallate (GCG) (1.8%), gallocatechin (GC) (1.8%), catechin (1.2%), and gallic acid (0.2%) (Slivova et al. 2005).

Green tea polyphenols demonstrate beneficial effects in different pathological conditions including obesity, diabetes and cancer. Polyphenols contained in the green tea were also found to inhibit tumor growth and invasion of cancers such as leukemia, those of prostate, lung, liver, and breast (Dreosti et al. 1997; Isemura et al. 1993) (Sartippour et al. 2001). In vitro studies using human MDA-MB-231 and MCF-7 breast cancer cells showed downregulation of MMP-2 and -9, EGFR and upregulation of TIMP-1 and -2, involvement of FAK/ERK/NF-κB signaling pathways with concomitant inhibition of cellular invasion (Farabegoli et al. 2011; Sen et al. 2010). Aqueous extract of green tea induced apoptosis and inhibited cell proliferation, migration and invasion in metastasis-specific mouse mammary carcinoma 4 T1 cells in vitro. Green tea extract was effective in vivo in decreasing tumor weight and significantly reduced lung and liver metastases incidence in female BALB/c mice bearing 4 T1 tumors (Luo et al. 2014). In vivo, green tea polyphenols inhibited the development and progression of lung, prostate, esophagus, stomach, intestine, skin, and other cancers (Katiyar & Mukhtar 1996; Yang et al. 2002). The induction of apoptosis by green tea polyphenols was found to be driven by mitochondria-targeted, caspase 3-executed mechanism (Hsu et al., 2003). The anti-invasive properties of the green tea polyphenols in breast cancer might be a result of preventing the formation of molecular complexes controlling cell adhesion and migration, specifically inhibition of activator protein-1 (AP-1) and NF-κB and consequent suppression of uPA secretion (Slivova et al. 2005).

Epidemiological studies, though inconclusive, suggest possible cancer preventive action of the green tea polyphenols. Nevertheless the beneficial effect of tea consumption for cancer prevention or progression is doubtful. In order to reach sufficient serum concentrations, high doses of polyphenols consumption are needed. Still, regular consumption of green tea has been associated with better prognosis in breast cancer patients (Nakachi et al. 1998) and possibly a decreased risk of recurrence (Inoue et al. 2001).

#### Epigallocatechin gallate (EGCG)

EGCG is the ester of epigallocatechin and gallic acid and it is the most abundant polyphenol in the green tea. In addition to green tea, EGCG is present in trace amounts in apples, plums, onions, hazelnuts, pecans, etc.

**Fig. 2:**
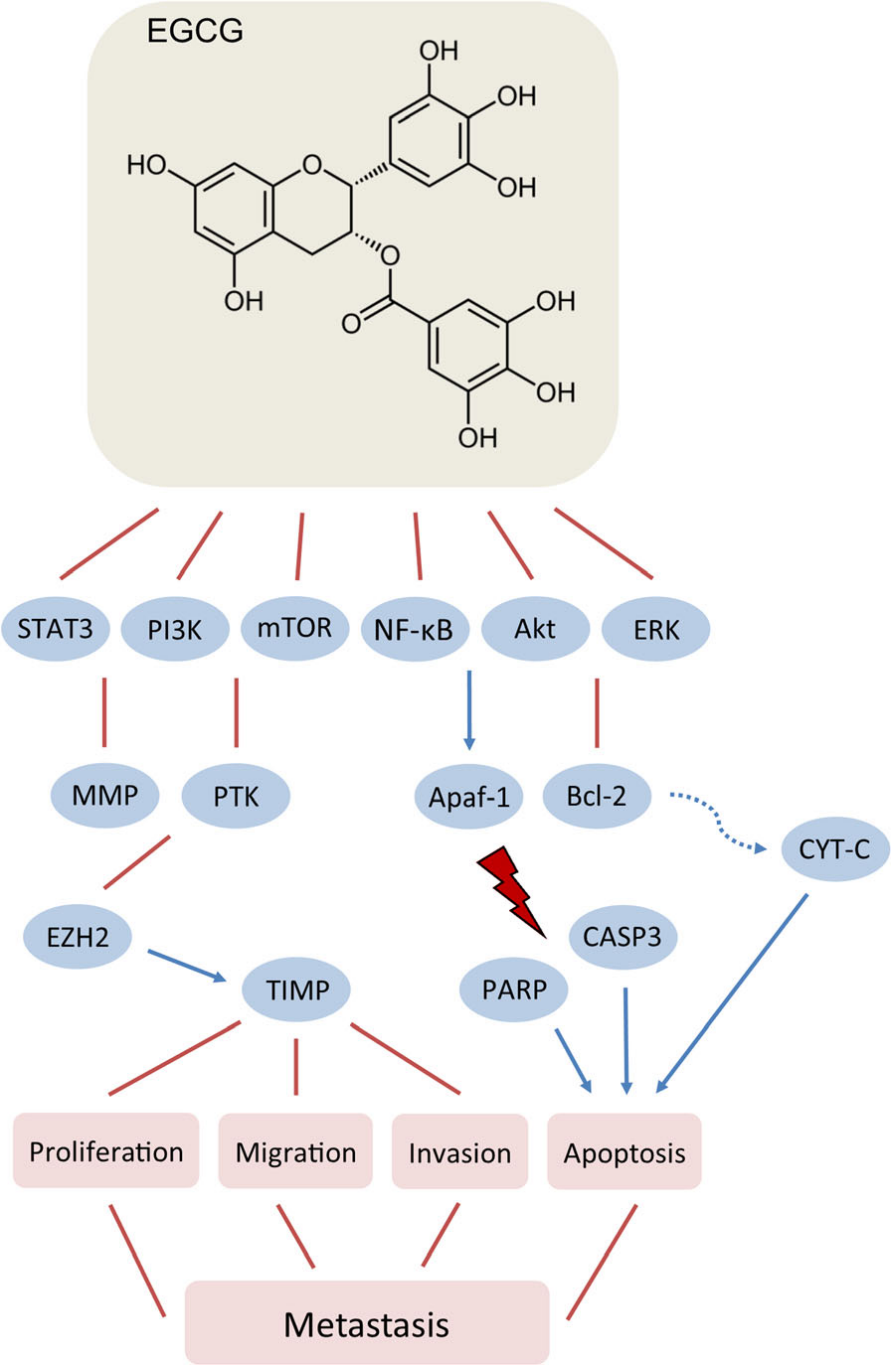
Effects of epigallocatachin gallate (EGCG) on breast cancer metastasis

Experimental data demonstrate that EGCG inhibits tumor cell proliferation, adhesion and invasion and induces apoptosis in variety of cancers including those of the breast (Fig. 2) (Ahmad et al. 1997; Yang et al. 2009; Shammas et al. 2006). Treatment of 4 T1 cells with EGCG decreases Bcl-2 expression and mitochondrial disruption thus releasing cytochrome C as well as upregulating Apaf-1, leading to the cleavage of caspase 3 and poly [ADP-ribose] polymerase (PARP) proteins (Baliga et al. 2005). In the same study, oral administration of green tea polyphenols to 4 T1-xenografted BALB/c mice resulted in reduction of tumor growth and lung metastasis incidence. The 67-kDa laminin receptor (67LR) has been identified as an essential cell surface target for EGCG action (Tachibana et al. 2004; Umeda et al. 2008). The mechanism of the tumor-suppressive and anti-metastatic actions of EGCG is a result of involvement of Akt/eNOS/NO/cGMP/PKCδ signaling cascade (Kumazoe et al. 2013). Similarly to other polyphenolic compounds, the effect of EGCG in cancer cells is pleiotropic. It inhibits the activities of PTKs (EGFR, FGFR, PDGFR, HER2/neu tyrosine kinases) and Akt kinase (Liang et al. 1997; Pianetti et al. 2002) via STAT3, PI3K, mTOR, and NF-κB signaling pathways (Masuda et al. 2002; Van Aller et al. 2011). Results from in vitro study by using MDA-MB-231 cells demonstrated that EGCG modulates cell matrix adhesion molecules and growth factor receptors through FAK/ERK signaling pathway mechanism (Sen & Chatterjee 2011). IGCG also inhibits the expression and activities of MMP-2 and -9 (Sen et al. 2009; Yang et al. 2005; Sen et al. 2010) (Farabegoli et al. 2011), and this seems to be the main driver for its anti-metastatic actions (Yang & Wang 1993). The inhibition of MMPs can be explained by the fact that EGCG suppresses FAK, PI3K, and ERK which further leads to downregulation of EGF (Sen & Chatterjee 2011). In addition, the suppression of MMPs involves epigenetic induction of TIMP-3 levels through inhibition of the enhancer of zeste homolog 2 (EZH2) and class I histone deacetylases (HDACs) (Deb et al. 2014). Short-term supplementation with the active compounds in green tea in men with prostate cancer showed that EGCG significantly reduces serum levels of VEGF (McLarty et al. 2009). Based on experimental data, it appears that plasma concentrations of EGCG comparable to those observed in regular green tea consumers are sufficient to inhibit MMPs and thus to affect negatively the invasion potential and metastasis in breast cancer patients (Garbisa et al. 2001).

In addition of suppressing tumor growth, ECGC was found to modulate tumor microenvironment by reducing TAM infiltration (Jang et al. 2013). In the same study, ex vivo incubation of TAM with exosomes from ECGC-treated mouse mammary tumor 4 T1 cells skewed macrophages from tumor-promoting M2-like to tumor-inhibitor M1-like phenotype (Jang et al. 2013). Further, EGCG targets tumor microenvironment by preventing and reversing the advancement of fibroblast-mediated effects by inhibiting signaling cascades downstream of TGFβ (Gray et al. 2014).

### Other phyto-polyphenols

Along with the above discussed phyto-polyphenols, a number of other compounds have been investigated for their anti-carcinogenic properties, including anti-metastatic actions. Plants rich in these polyphenolic compounds have been used for centuries in culinary and traditional medicine.

#### Kaempferol

Kaempferol (3,5,7-Trihydroxy-2-(4-hydroxyphenil)-4*H*-chromen-4-one) is a naturally occurring flavonol in broad range of plants from *Pteridophyta*, *Pinophyta* and *Angiospermae* divisions. Among the commonly consumed foods containing kaempferol are grapes, green tea, apples, tomatoes, potatoes, onions, broccoli, squash, Brussels sprouts, cucumbers, lettuce, green beans, peaches, blackberries, raspberries, spinach, etc. Kaempferol is actively absorbed in the small intestine and can be found in the plasma in nanomolar concentrations (Calderón-Montaño et al. 2011). This polyphenol is easily metabolized in the liver and is delivered to various other organs in the form of glucuronides and sulfoconjugates (Calderón-Montaño et al. 2011).

**Fig. 3:**
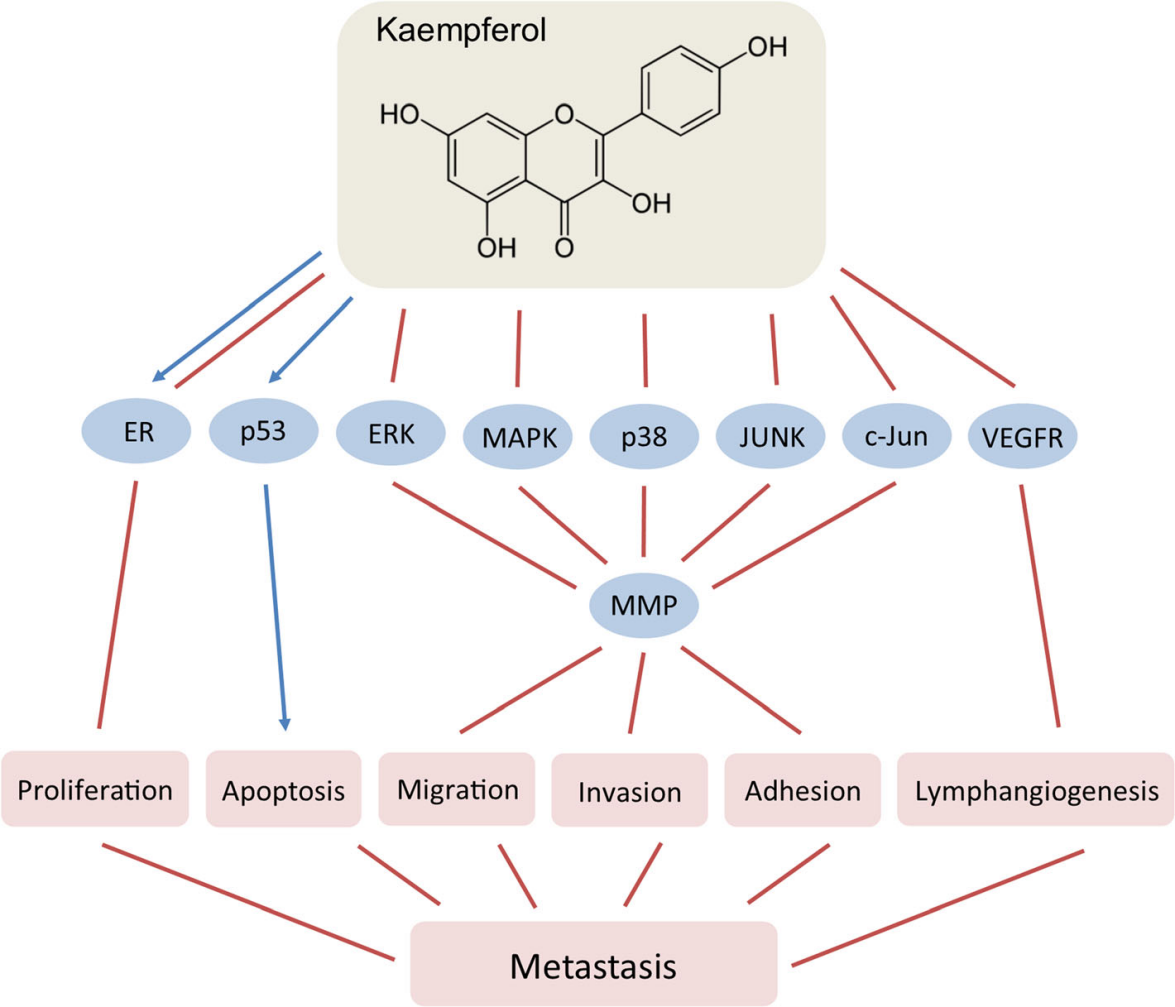
Effects of kaempferol on breast cancer metastasis

To date, kaempferol has been shown to exert a variety of effects including antioxidant, anti-inflammatory, anti-microbial, anxiolytic, anti-allergic as well as anti-carcinogenic and cancer preventive activities (Calderón-Montaño et al. 2011). Multiple in vitro and in vivo studies demonstrated that kaempferol has pleiotropic effects in cancer targeting cancer cell proliferation, apoptosis and mobility, tumor growth, angiogenesis and metastasis (Fig. 3) (Kim & Choi 2013; Calderón-Montaño et al. 2011; Boam 2015; Srinivas 2015). Kaempferol is an endocrine-disruptor that influences the activity of ER, having both, estrogenic and anti-estrogenic properties (Calderón-Montaño et al. 2011). This makes kaempferol potentially useful in ER-positive breast cancers, where it suppresses tumor growth by ER-dependent mechanism (Oh et al. 2006).

Kaempferol interacts with major signaling pathways such as ERK1/2 (Aiyer et al. 2012), MAPK (Li et al. 2015), and p53 (Calderón-Montaño et al. 2011), and is a potential anti-metastasis agent. It inhibits the invasion, adhesion, and migration of U-2 osteosarcoma cells (Chen et al. 2013). Anti-metastatic effects of kaempferol were observed in SCC4 oral cancer cells where it downregulated MMP-2 and TIMP-2 mRNA and protein expression by suppressing c-Jun activity (Lin et al. 2013). Recent study found that kaempferol inhibits MDA-MB-231 breast cancer cell adhesion, migration and invasion, and reduces lung metastasis incidence in mice (Li et al. 2015).

The mechanisms behind the anti-metastatic effects of kaempferol include supression of MMPs (MMP-2 and MMP-9) and uPA expression and activity via ERK, p38, JNK, and MAPK signaling (Chen et al. 2013). Kaempferol inhibits the translocation of the MAPK upstream regulator PKCδ from the cytoplasm to the plasma membrane where it is physiologically active, thus suppressing MAPK signaling pathway (Li et al. 2015). Another mechanism by which kaempferol suppress metastasis is by inhibiting VEGF production as demonstrated in ovarian cancer OVCAR-3 cells in vitro *(*Luo et al. 2008*)*. In the same cell line, kaempferol was also shown to downregulate cMyc and promote apoptosis (Luo et al. 2010). Additionally, kaempferol inhibits lymphangiogenesis, which is an integral step in the metastatic process. It reduces the density of tumor-associated lymphatic vessels as well as the incidence of lymph node metastases in breast cancer xenograft models in a VEGFR2/3 kinase manner (Astin et al. 2014).

#### Curcumin

Curcumin (*s.* diferuloylmethane, E100 (Natural Yellow 3)) ((1*E*,6*E*)-1,7-Bis(4-hydroxy-3-methoxyphenyl)-1,6-heptadiene-3,5-dione) is a natural diarylheptanoid polyphenol derived from turmeric plant (*Curcuma longa*) belonging to the ginger family (*Zingiberaceae*). Turmeric is common ingredient of the traditional Indian cuisine (main ingredient of curry) as well as it is used worldwide as a food additive for coloring (bright-yellow agent E100). Additionally, turmeric is known for its medicinal properties. Powdered turmeric underground stems (rhizomes) have been used for more than 6000 years for treating broad range of conditions related to inflammation, allergies, parasitic infections, respiratory diseases, diabetes, neurodegenerative diseases and many others. Turmeric-derived curcumin has also well established anti-carcinogenic activities on cell transformation, proliferation, apoptosis, survival, invasion, metastasis, adhesion as well as angiogenesis. The anti-carcinogenic effects of curcumin have been demonstrated in different studies on hematogenous, multiple myeloma, glioblastoma, skin, head and neck, lung, colon, prostate, breast, and other types of cancer (Bachmeier et al. 2007; Kuo et al. 1996; Sung et al. 2009; Dhandapani et al. 2007; Limtrakul et al. 1997; Wilken et al. 2011; Moghaddam et al. 2009; Chen et al. 1999; Kawamori et al. 1999; Johnson & Mukhtar 2007; Chendil et al. 2004; Mehta et al. 1997; Huang et al. 1998; Killian et al. 2012).

Curcumin is poorly metabolized and extensively excreted. It can be found in low concentrations in plasma and variety of tissues (Anand et al. 2007). Despite its lower bioavailability, curcumin in low concentrations has been shown to possess toxicity selectively to cancer, but not to untransformed cells (Syng-Ai et al. 2004). For example, experimental data showed that human multidrug-resistant breast cancer MCF-7/TH cells are approximately 3.5-fold more sensitive to curcumin than the non-carcinogenic epithelial MCF-10A cells (Ramachandran & You 1999).

The anti-carcinogenic properties of curcumin are pleiotropic and are based on its effects on both, the tumor cells and the tumor microenvironment. For example, curcumin can modulate inflammatory pathways and tumor progression and metastasis, affecting tumor cell survival, proliferation, and invasiveness (Gupta et al. 2010). Curcumin as well as other plant-derived natural polyphenols such as EGCG or resveratrol, induce epigenetic changes (inhibition of DNA methyltransferases (DNMTs), regulation of histone acetyltransferases (HATs) and HDACs, or microRNA modulation) (Gonwa et al. 1989) that lead to suppression of EMT and metastasis (Bandyopadhyay 2014; Bachmeier et al. 2007; Kunnumakkara et al. 2008).

**Fig. 4:**
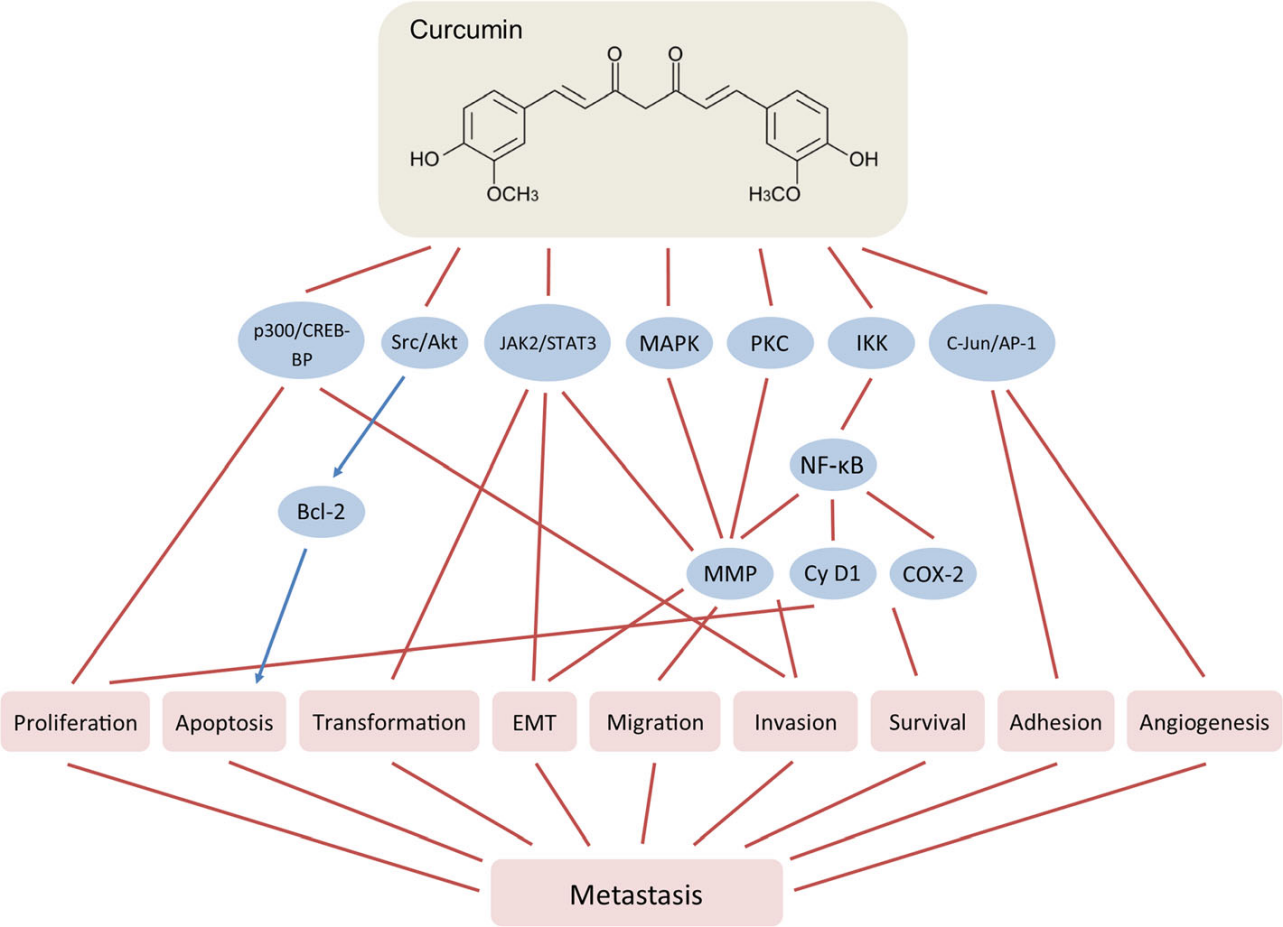
Effects of curcumin on breast cancer metastasis

The anti-metastatic action of curcumin involves inhibition of MMP-2, − 9, and MT1-MMP (Ohashi et al. 2003; Kim et al. 2012) (Fig. 4). Curcumin acts as specific supressor of p300/CREB-binding protein and affects major signalling pathways, protein tyrosine kinases and cytokines such as MAPK (Kim et al. 2012), JAK2/STAT3, Src/Akt (Saini et al. 2011), c-Jun/AP-1 (Collett & Campbell 2004), PKC (Garg et al. 2008), sonic hedgehog (Elamin et al. 2010), CXCL1 and 2 (Killian et al. 2012), etc. It also inhibits HDACs 1, 3, and 8 and HATs enzyme activities and modulates chromatin modification (Balasubramanyam et al. 2004; Reuter et al. 2011). In addition, curcumin suppresses NF-κB signaling by negative modulation of IKK, either directly or through action of its upstream activators (Bharti et al. 2003; Jobin et al. 1999), preventing in such a way phosphorylation of IκB (Plummer et al. 1999). Curcumin abolishes the DNA binding of NF-κB and inhibits reporter gene expression in H1299 non-small cell lung carcinoma cell line, thus downregulating MMP-9 activation (Shishodia et al. 2003). In mice, where MDA-MB-231 breast cancer cells were injected intracardiac, oral curcumin administration significantly reduced the number of lung metastases (Bachmeier et al. 2007). This effect was most likely a result of inhibition of NF-κB activity and transcriptional downregulation of AP-1 and downregulation of cyclin D1, COX-2, and MMP-9, which further leads to inhibition of the breast cancer cell metastasis (Aggarwal et al. 2005; Kim et al. 2012).

Chronic inflammation is considered to be a major factor in tumor progression. For example, chronic prostatitis, chronic obstructive pulmonary disease, inflammatory bowel disease or chronic pancreatitis – all represent risk factors for developing prostate, lung, colon or pancreatic cancer. Curcumin inhibits chronic inflammation by disrupting the feedback loop between NF-κB and the pro-inflammatory cytokines, CXCL-1 and -2 (reviewed by Bandyopadhyay (Bandyopadhyay 2014)).

By inhibiting NF-κB signaling, curcumin suppresses metastasis in the very early stages of EMT. In lipopolysaccharide (LPS)-induced EMT in MCF-7 and MDA-MB-231 cells, curcumin downregulated the expression of vimentin and upregulated those of E-cadherin as well as inhibited LPS-induced morphological transformation of the cells through inactivation of NF-κB-SNAIL signaling pathway (Huang et al. 2013).

Curcumin acts also as a phytoestrogen (Bachmeier et al. 2010). The anti-proliferative effects of curcumin were found to be estrogen-dependent in ER-positive MCF-7 counteracting the estrogen responsive element (ERE)-CAT activities of estradiol (Shao et al. 2002). HER2/neu-positive or tamoxifen-resistant breast tumors are associated with specific microRNA signature, including overexpression of miR-181 (Miller et al. 2008; Lowery et al. 2009). In breast cancer, curcumin was shown to inhibit metastasis by inducing the expression of miR-181b and downregulatinng those of CXCL-1 and -2 (Kronski et al. 2014).

A variety effects on tumor microenvironment were described after curcumin treatment. In colon cancer, curcumin interacts with the stromal fibroblasts in the colon tumor microenvironment thus suppressing their crosstalk with CSCs (Buhrmann et al. 2014). Treatment with curcumin-polyethylene glycol conjugate (an amphiphilic curcumin-based micelle) suppressed the percentage of myeloid-derived suppressor cells (MDSCs), which was suggested to be the reason behind the observed inhibition of Treg and the activation of the effector T-cells (Lu et al. 2016). Combination of curcumin and ECGC inhibits colorectal carcinoma microenvironment-induced angiogenesis by activating JAK/STAT3/IL-8 signaling pathway (Jin et al. 2017). Curcumin downregulates the expression of VEGF as shown in prostate cancer cells (Gupta et al. 2013) and blocked IL-1 and VEGF expression in chondrosarcoma cells (Kalinski et al. 2014).

Currently, curcumin is an object of more than 120 clinical trials evaluating its effects against different maladies including cancer.

#### Honokiol

Honokiol is a biphenolic lignan with bioactive para-allyl and ortho-allyl phenolic groups, a product of *Magnolia sp.* (*M. biondii*, *M. obovate*, and *M. officinalis*) that demonstrates promising actions on tumor metastases. Bark or seed cones of magnolia plants has been used for centuries in the traditional Asian medicine for its anti-inflammatory, antithrombotic, anxiolytic, antidepressant, antispasmodic, antioxidant, and antibacterial effects and its protective action against hepatotoxicity, neurotoxicity and angiopathy (Fried & Arbiser 2009; Lee et al. 2011). The anti-carcinogenic activities of honokiol range from tumor suppression, pro-apoptotic and anti-angiogenic effects, and inhibition of cancer metastasis incidence by effects on tumor proliferation, migration and invasion. These properties have been demonstrated in various cancer types such as sarcoma (Nagase et al. 2001; Su et al. 2013), multiple melanoma (Ishitsuka et al. 2005), leukemia (Hirano et al. 1994; Hibasami et al. 1998; Battle et al. 2005), lung (Yang et al. 2002; Singh & Katiyar 2013), skin (Konoshima et al. 1991), pancreas (Bai et al. 2003), ovary (Li et al. 2008), prostate (Shigemura et al. 2007), colorectal (Wang et al. 2004), breast (Nagalingam et al. 2012; Avtanski et al. 2014), and other cancers (Nagase et al. 2001; Garcia et al. 2008; Deng et al. 2008; Chen et al. 2011; Chang et al. 2013). One important characteristic of honokiol is that it easily crosses the blood-brain barrier and achieves significant serum concentrations because of its hydrophobic and lipophilic properties (Wang et al. 2011; Lin et al. 2012; Woodbury et al. 2013).

**Fig. 5:**
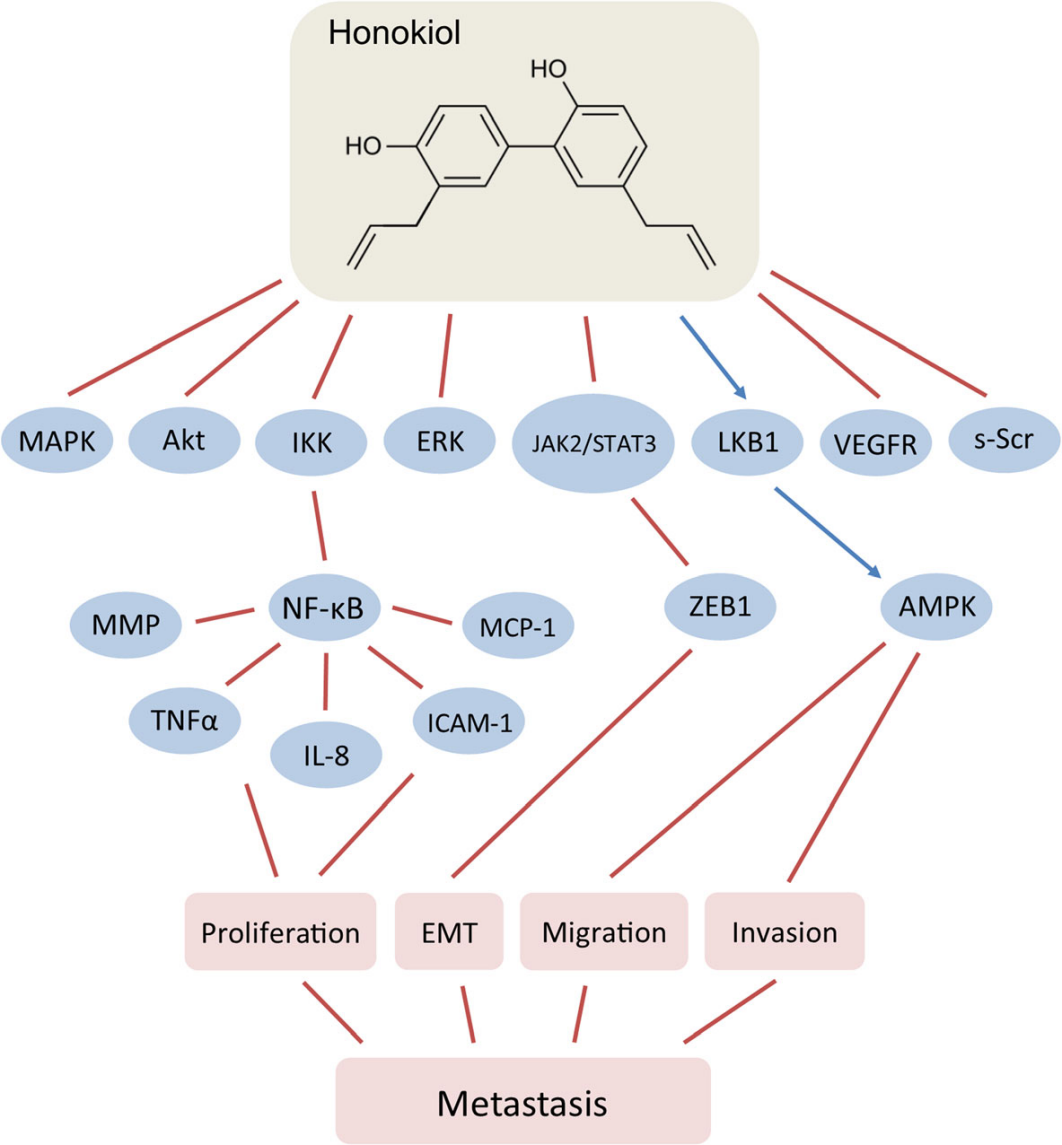
Effects of honokiol on breast cancer metastasis

Honokiol has pleiotropic effects in the cells (Fig. 5), including modulation of NF-κB (Tse et al. 2005; Lee et al. 2005; Ahn et al. 2006; Sheu et al. 2008; Arora et al. 2011), MAPK (Kim et al. 2012; Zhang et al. 2014), STAT3 (Rajendran et al. 2012; Avtanski et al. 2014), Akt [238,], VEGF (Wen et al. 2015), ERK (Zhu et al. 2014; Yeh et al. 2016), s-Scr (Park et al. 2009), and other major signaling pathways (Fried & Arbiser 2009). For example, in SVR angiosarcoma cells, honokiol induces apoptosis by suppressing the phosphorylation of ERK, Akt, and c-Src (Bai et al. 2003). In addition to its anti-proliferative properties, honokiol inhibits the migration and tube formation of human umbilical vein endothelial cells (HUVECs) and suppresses angiogenesis in zebrafish angiogenesis model (Zhu et al. 2011). Honokiol downregulates IKK activation and thus inhibits NF-κB signaling pathway and MMP-9, TNFα, IL-8, ICAM-1, and MCP-1 expression (Tse et al. 2005; Lee et al. 2005; Ahn et al. 2006; Sheu et al. 2008). It also inhibits the migration and invasion of MCF-7 and MDA-MB-231 cells by upregulating the activity of liver kinase B1 (LKB1) leading to activation of AMP-activated protein kinase (AMPK) (Nagalingam et al. 2012). In vivo, honokiol inhibited tumor growth of MDA-MB-231 cells-xenografted nude mice by blocking breast cancer cellular proliferation (Nagalingam et al. 2012). Our in vitro and in vivo studies revealed that honokiol inhibits EMT of breast cancer cells by suppressing STAT3 signaling resulting in repression of ZEB1 expression and its recruitment on the E-cadherin promoter (Avtanski et al. 2014). Honokiol modulated microRNA profile in the breast cancer cell, specifically amplifying miR-34a expression in a STAT3-dependent manner, inhibiting Wnt1-metastatic-associated protein 1 (MTA1)-β-catenin signaling axis (Avtanski et al. 2015a). The mechanism behind the effects of honokiol on EMT and breast cancer migration involves induction of SirT1, SirT3 and miR-34a expression and cytoplasmic localization of LKB1 (Avtanski et al. 2015b).

Aside from directly targeting tumor cells, honokiol was also demonstrated to have effects on tumor microenvironment. Honokiol decreased desmoplasia in pancreatic tumor xenografts, as characterized by reduced secretion of extracellular matrix protein (collagen I) and suppressed myofibroblast marker α-smooth muscle actin (α-SMA) immunostaining (Averett et al. 2016). Findings from the same study revealed an inhibitory effect of honokiol on C-S-C chemokine receptor type 4 (CXCR4) signaling, which is known to play an important role in the crosstalk between the tumor and the stromal cells.

## Conclusions

Nature is abundant in chemicals with potential therapeutic effects that are worth studying. A variety of polyphenols from plant origin demonstrate pleiotropic therapeutic properties against a broad range of pathological conditions, including different types of cancer. Such polyphenolic compounds can be viewed as promising candidates for supplements to the traditional cancer prevention and treatment modalities as well as a basis for designing novel synthetic drugs. Naturally derived plant polyphenols have been demonstrated to inhibit metastasis initiation and progression by targeting both, cancer cells and cancer microenvironment. Novel strategies for targeting metastasis aim to modulate the levels of specific microRNAs that play a role in the transformation of the malignant cells. This approach could be used against CSCs or cells undergoing EMT that are typically drug resistant (Li et al. 2010). Importantly, some phyto-polyphenolic compounds have been shown to exert beneficial effects through direct modulation of specific microRNAs at low concentrations.

Natural polyphenolic compounds are usually characterized by low level of toxicity, but main disadvantage is their poor bioavailability and weak resorption reaching. In this regard, new strategies for target-specific delivery have been experimentally developed and proven to be effective. Recent advances in nano-medicine open the doors for the development of vehicles for drug delivery with long-circulation that can be used to target transformed cells. Polyphenolic compounds administered by traditional methods are not always effective because of they are poorly absorbed and extensively excreted. But the chemopreventive efficacy of these polyphenols can be significantly improved by encapsulating them into nonoparticles. Thus, integration of various disciplines such as biochemistry, molecular biology, chemistry, and nanotechnology could contribute to the development of novel therapies against breast cancer methastasis.

This paper is dedicated to the memory of Rumiana Cherneva, who lost the battle with breast cancer.

## Abbreviations

67LR: 67-kDa Laminin Receptor
AMF: Autocrine Motility Factor
AMPK: 5’ AMP-Activated Protein Kinase
AP-1: Activator Protein-1
BCL: B-Cell Lymphoma
C/EBPα: CCAAT-Enhancer Binding Protein-Alpha
CAA: Cancer-Associated Adipocyte
CAF: Cancer-Associated Fibroblast
CAM: Cell Adhesion Molecule
CCL2: Chemokine (C-C motif) Ligand 2
COX: Cyclooxygenase
CSC: Cancer Stem Cells
CXCR4: C-S-C chemokine Receptor type 4
DNMT: DNA Methyltransferase
EC: Epicatechin
ECG: Epicathechin Gallate
ECM: Extracellular Matrix
EGC: Epigallocatechin
EGCG: Epigallocatechin-3-Gallate
EGF: Epidermal Growth Factor
EGFR: Epidermal Growth Factor Receptor
EMT: Epithelial-to-Mesenchymal Transition
ER: Estrogen Receptor
ERE: Estrogen Responsive Element
ERK: Extracellular-Signal-Regulated Kinase
EZH: Enhancer of Zeste Homolog
FAK: Focal Adhesion Kinase
FGFR: Fibroblast Growth Factor Receptor
FOXO1: Forkhead Box O1 Protein
GC: Gallocatechin
GCG: Gallocatechin Gallate
HAT: Histone Acetyltransferase
HDAC: Histone Deacetylase
HER2: Human Epidermal Growth Factor Receptor 2
HUVEC: Human Umbilical Vein Endothelial Cells
IFNγ: Interferon-Gamma
IkB: Inhibitors of kappa B
IKK: Inhibitors of kappa B Kinase Kinases
IL: Interleukin
LKB1: Liver Kinase B1
LPS: Lipopolysaccharide
α-SMA: Alpha-Smooth Muscle Actin
MAPK: Mitogen-Activated Protein Kinases
MAPKK: Mitogen-Activated Protein Kinase Kinase
M-CSF: Macrophage Colony-Stimulating Factor
MDSC: Myeloid-Derived Suppressor Cells
MEKK: MAPK/ERK Kinase Kinase
miR: MicroRNA
MMP: Matrix Metalloproteinase
MSP: Motility-Stimulating Protein
mTOR: Mammalian Target of Rapamycin
NF-κB: Nuclear Factor Kappa-Light-Chain-Enhancer of Activated B Cells
NIK: Nuclear Factor Kappa-Light-Chain-Enhancer of Activated B Cells-Inducing Kinase
NFKBIA: Nuclear Factor of Kappa Light Polypeptide Gene Enhancer in B-Cells Inhibitor-Alpha
ODC: Ornithine Dacarboxylase
PAI: Plasminogen Activator Inhibitor
PARP: Poly [ADP-Ribose] Polymerase
PDGFR: Platelet-Derived Growth Factor Receptor
PI3K: Phosphatidylinositol-3-Kinase
PKC: Protein Kinase C
PR: Progesterone Receptor
RAF: Rapidly Accelerated Fibrosarcoma Kinase
ROS: Reactive Oxygen Species
RTK: Receptor Tyrosin Kinase
SERM: Selective Estrogen Receptor Modulators
TEC: Tumor Endothelial Cells
TIL: Tumor-Infiltrating Lymphocyte
TIMP: Tissue Inhibitors of Metalloproteinases
TNFα: Tumor Necrosis Factor-Alpha
uPA: Urokinase-Type Plasminogen Activator
VEGF: Vascular Endothelial Growth Factor
XIAP: X-linked Inhibitor of Apoptosis Protein
